# Enhancing abscisic acid production in *Botrytis cinerea* through metabolic engineering based on a constitutive promoter library

**DOI:** 10.1016/j.synbio.2024.12.004

**Published:** 2024-12-20

**Authors:** Ling-Ru Wang, Ji-Zi-Hao Tang, Shu-Ting Zhu, Na Wu, Zhi-Kui Nie, Tian-Qiong Shi

**Affiliations:** a School of Food Science and Pharmaceutical Engineering, Nanjing Normal University, Nanjing, 210023, China; b College of Marine and Bioengineering, Yancheng Institute of Technology, Yancheng, China

**Keywords:** Abscisic acid, *B. cinerea*, Promoter library, Cofactor supply, Metabolic engineering

## Abstract

Abscisic acid (ABA) is an important plant growth regulator with broad applications in agriculture, forestry, and other fields. Currently, the industrial production of ABA primarily relies on microbial fermentation using *Botrytis cinerea*, but its genetic toolbox is limited. To address this, we first screened 10 strong constitutive promoters from the genome of *B. cinerea* through transcriptomic analysis. The expression levels of the promoters covered a range of 3–4 orders of magnitude according to the measured β-glucuronidase activity. Subsequently, four promoters of different strength were used to balance the cofactor supply in *B. cinerea*. Overexpression of NADH kinase using the medium-strength promoter *Pef1a* significantly enhanced ABA production, resulting in a 32.26 % increase compared to the control. Finally, by combining promoter engineering with a push-pull strategy, we optimized the biosynthesis of ABA. The recombinant strain Pthi4:hmgr-Pef1a:a4, overexpressing HMGR under the *Pthi4* promoter and Bcaba4 under the *Pef1a* promoter, achieved an ABA titer of 1.18 g/L, a 58.92 % increase. To our best knowledge, this is the first constitutive promoter library suitable for *B. cinerea*, providing important tools for the industrial production of ABA.

## Graphical abstract

**Figure:**
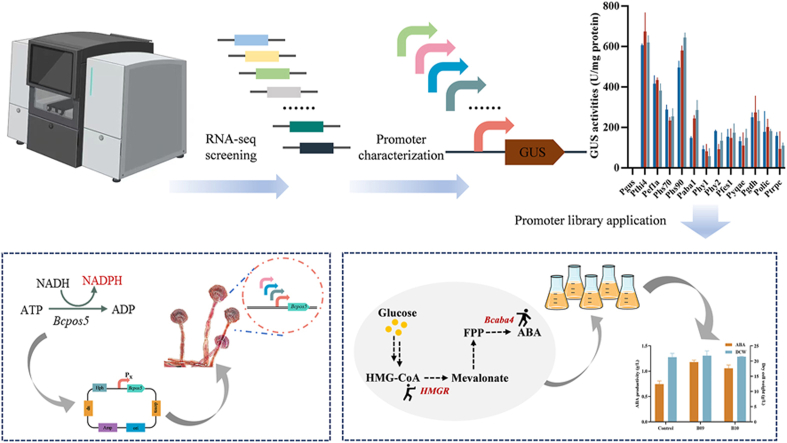

## 1 Introduction

Abscisic acid (ABA) is a sesquiterpene compound and one of the five major plant growth regulators. Known for its physiological functions in enhancing plant stress tolerance as well as regulating plant growth and development, ABA has been widely applied in agricultural production [1,2]. In addition to higher plants, ABA is also found in microalgae, fungi, bacteria, and animals [[3], [4], [5], [6], [7]]. Research has demonstrated that ABA can regulate glucose homeostasis in mammals and reduce symptoms of diseases such as inflammation and malaria, indicating significant potential for medical applications [8,9]. The economic feasibility of large-scale ABA production is crucial for unlocking its application potential. However, ABA produced through chemical synthesis is often a racemic mixture, which significantly reduces its biological activity and complicates industrial-scale production [10]. Therefore, microbial fermentation has become the primary method for the industrial production of ABA.

*Botrytis cinerea* is a common pathogenic fungus that can infect over 1400 plant species, including many economically important crops, posing a serious threat to agricultural production [11]. Due to its significant economic impact, *B. cinerea* has become a model microorganism for studying necrotrophic plant pathogens [12]. Additionally, *B. cinerea* has promising industrial applications, as it is capable of synthesizing diverse products such as the sesquiterpene ABA and its derivatives, the phytotoxin botrydial, and glucanase [[13], [14], [15], [16]]. Notably, *B. cinerea* has an exceptional ability to synthesize ABA, surpassing other producers, and its fermentation has been industrialized [17]. However, challenges such as high production costs and low productivity still remain.

The emergence of synthetic biology has provided opportunities for the rational modification of *B. cinerea* to enhance ABA production. However, a comprehensive genetic toolbox, including promoters, terminators, and markers, is crucial for metabolic engineering [18]. Among these, promoters are key factors controlling gene expression levels and are essential for regulating metabolic pathways to increase product synthesis [19]. Currently, the commonly used promoters in *B. cinerea* are limited to the *oliC* and *trpC* promoters from *Aspergillus nidulans* [20]. Additionally, weak promoters such as the actin promoter *PactA* from *B. cinerea* and the glyceraldehyde-3-phosphate dehydrogenase promoter *Pgpd* from *A. nidulans* have also been used in genetic engineering studies of *B. cinerea* [21,22]. However, there is still a lack of exploration of strong promoters for genetic engineering in *B. cinerea*, which restricts the progress of its genetic engineering. Therefore, the development of a strong promoter library suitable for *B. cinerea* is particularly important. Since the strength of promoters may be significantly affected by the genetic background of the host strain, identifying strong promoters from the endogenous genes of *B. cinerea* may be a more effective approach. Transcriptome sequencing has become a powerful tool for identifying different types of endogenous promoters in microorganisms. Wang et al., used transcriptome data from *Lactococcus cremoris* and characterization results of the β-glucuronidase (GUS) reporter gene to develop a library containing eight constitutive promoters with varying strengths [23].

Although *B. cinerea* has intrinsic advantages for producing ABA, there is currently little research on metabolic engineering and genetic modification tools for this strain, which limits its potential for industrial application. To overcome these restrictions, this study first utilized transcriptomics and RT-qPCR to screen strong endogenous constitutive promoters in *B. cinerea* and characterized their expression strength using a reporter gene, resulting in a stable library of constitutive promoters. Next, the promoter library was used as the basis for a metabolic engineering strategy to tune the cofactor supply levels and ABA biosynthesis pathway, thereby enhancing the production capacity of *B. cinerea*.

## 2 Material and methods

### 2.1 Strains and plasmids

The parental strain used in this study was *B. cinerea* NNU-1. *Escherichia coli* DH5α was used as the cloning host. All strains, plasmids, and DNA primers utilized in this study are presented in supplementary material. To construct the plasmids used for promoter strength determination, the corresponding promoter fragments were amplified from genomic DNA of *B. cinerea* and inserted into the pUC-GUS vector, which contains up and downstream fragments of nitrite reductase (*niiA*), a hygromycin expression cassette, and GUS coding sequence. The construction was performed using the ClonExpress MultiS One Step Cloning Kit purchased from Vazyme (Nanjing, China). Additionally, plasmids for gene overexpression were constructed by amplifying *Bcpos5* (NCBI reference: XM_024695525.1) and *HMGR* (NCBI reference: XM_001559909.2) gene fragments from the genome of *B. cinerea* and inserting them into pUC-△niiA vector with *niiA* flanking sequences and hygromycin resistance cassette. The corresponding promoters, target genes, and pUC-△niiA were ligated using the Kit. Similarly, the corresponding promoters and the *Bcaba4* (NCBI reference: XM_001553919.2) gene fragment were amplified from genomic DNA of *B. cinerea* and inserted into the pUC-△niaD vector, which contains the up- and downstream fragments of the nitrate reductase (*niaD*) gene and a G418 resistance expression cassette.

### 2.2 Culture conditions

The seed culture medium contained glucose 30 g/L, wheat bran 70 g/L, MgSO4 0.5 g/L, KH_2_PO_4_ 0.5 g/L, and NH_4_NO_3_ 0.3 g/L. The fermentation medium contained glucose 80 g/L, yeast extract 10 g/L, wheat bran 10 g/L, MgSO4 1 g/L, KH_2_PO_4_ 1 g/L, and vitamin B_1_ 0.03 mg/L. *B. cinerea* preserved in 20 % glycerol at −80 °C was streaked onto potato dextrose agar (PDA) and cultured for 8–10 days. Afterwards, the fungal mycelium was washed off and placed in the seed medium, then cultured at 26 °C and 180 rpm for 48 h. Following this, it was inoculated into the fermentation medium at a 4 % inoculation rate and cultured for 8 days under the same temperature and agitation conditions.

### 2.3 RNA sequencing

*B. cinerea* was cultured at 26 °C and 180 rpm. After 24 and 96 h, the fungal mycelium was harvested. Total RNA was extracted using an RNA extraction kit purchased from Vazyme (Nanjing, China). The TruSeq™ RNA Sample Prep Kit (Illumina, San Diego, CA, USA) was used to construct the cDNA library, and the RNA-seq libraries were constructed by an Illumina NovaSeq6000 platform from Jinweizhi (Suzhou, China). Gene expression was calculated using HTSeq software, and the expression levels were determined based on FPKM values. For both the 24-h and 96-h time points, three replicate samples were taken. One sample with RNA quality unqualified for further analysis was obtained at the 96-h time point. A total of 234,692,916 paired end reads were obtained, with an average of 46,938,583 pure reads per sample. We previously performed whole-genome sequencing of *B. cinerea* NNU-1 and conducted high-throughput annotation of the biological functions of all genes in the genome using public databases such as the Kyoto Encyclopedia of Genes and Genomes (KEGG) and the Non-redundant Protein Database, predicting a total of 12,799 putative protein-coding genes. After aligning the reads to the genomic sequence, it was found that an average of 43,932,719 reads per sample (accounting for 92.28 % of the total clean reads) could be uniquely mapped to the genomic sequence. Genes with no significant differences in transcriptional levels (log2-FoldChange < ±1, q-value >0.05) were selected.

### 2.4 RT-qPCR

After RNA extraction as described above for RNA sequencing, cDNA synthesis was performed with the HiScript III RT SuperMix kit from Vazyme (Nanjing, China). RT-qPCR analysis was then conducted with QK Platinum SYBR Green Master Mix and a QuantStudio 3 Real-Time PCR System (Thermo Fisher Scientific, USA). Gene expression levels were determined by the 2^−ΔΔCT^ method and adjusted relative to β-actin expression levels. The utilized primer sequences are listed in supplementary material, and each experiment included three biological replicates.

### 2.5 Predictive analysis of the regulatory elements of promoters

The DNA sequences of 1500 bp upstream of the translation start site (TLS) were identified using the Berkeley Drosophila Genome Project website (https://fruitfly.org/seq_tools/promoter.html) to determine the putative transcription start site (TSS). Additionally, regulatory promoter elements for each gene (*i.e.*, TATA boxes and transcription factor binding sites) were identified using the Softberry website (http://www.softberry.com/berry.phtml?topic=index&group=programs&subgroup=promoter).

### 2.6 Protoplast transformation of *B. cinerea*

Mycelia were washed off from PDA plates and grown in liquid PD medium (PDA without agar) for 48 h. The culture was then transferred to fresh liquid PDA medium and incubated for an additional 18 h. The mycelia were filtered through sterile lens paper, and the collected mycelia were used for protoplast preparation. A mixed enzyme solution was prepared with 1 % driselase (Sigma-Aldrich, USA), 1 % lysing enzyme (Sigma-Aldrich, USA), and 0.7 mol/L NaCl. Then, 1 g of wet mycelia was mixed with 10 mL of the mixed enzyme solution, and the mixture was subjected to enzymatic digestion for 3 h. The enzymatic solution was filtered through sterile lens paper to remove mycelial debris, followed by centrifugation to collect the protoplasts. The protoplasts were subjected to two washes with pre-cooled STC buffer at 4 °C, then suspended in an appropriate amount of STC buffer, and adjusted to a final concentration of 10^7^-10^8^/mL.

For transformation 200 μL of the protoplast preparation was combined with 10–15 μg of DNA (maximum volume 10 μL), 50 μL of PEG4000 solution (25 % PEG 4000, 50 mM CaCl_2_, 10 mM Tris-HCl, pH 7.5), and 10 μg sgRNA targeting the *niiA* locus, mixed well and kept on ice for 20 min. Next, 1 mL of PEG4000 solution was introduced and left to stand at ambient temperature for 10 min, after which 2 mL of STC buffer was incorporated. After mixing, 160 μL of the mixture spread onto a PDA plate containing 0.6 M sucrose. After incubating for 16 h, the dish was overlaid with 9 mL of antibiotic-containing soft agar PDA at 42 °C. After incubation at 26 °C for 5–7 days colonies appeared and were subcultured on selective medium for 3 to 5 generations to isolate pure clones.

### 2.7 Enzyme activity assays

A series of GUS-expressing strains containing candidate promoters were collected after 24, 48, and 72 h of cultivation. GUS activity was analyzed with a quantitative GUS reporter detection kit purchased from Coolaber (Beijing, China). Additionally, GUS activity was measured in a series of GUS-expressing strains containing candidate promoters after 48 h of cultivation in fermentation media with glucose, xylose, sucrose, or fructose as the main carbon sources. The GUS activity was measured using the kit. In addition, NADPH concentration was measured with an NADP^+^/NADPH Quantification Kit (Biosharp, China) following the protocol provided by the supplier.

### 2.8 Measurement of ABA and DCW concentration

The fermentation broth was analyzed using liquid chromatography on an LC-20A instrument (Shimadzu, Japan) with a UV detector (SPD-20A) set to 260 nm to measure ABA content. A C18 column (VA952505-0, Agela, China) was used, and the column was kept at a temperature of 30 °C. The mobile phase consisted of 0.1 % formic acid mixed with 100 % methanol, operating at a flow rate of 0.3 mL/min. The dry cell weight (DCW) was determined using gravimetric analysis. Briefly, 50 mL of fermentation broth was centrifuged at 7000 *g* for 15 min to remove the supernatant, the pellet was washed twice with water, and then dried in a freeze-dryer to a constant weight.

## 3 Results and discussion

### 3.1 Identification of endogenous constitutive promoters in *B. cinerea* through transcriptome analysis

**Fig. 1:**
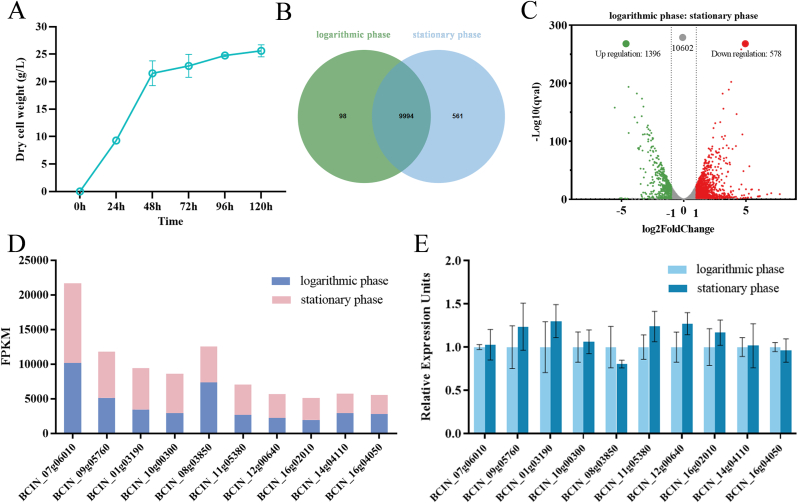
Identification of constitutive promoters in *B. cinerea* based on transcriptome analysis. (A) Growth curve of *B. cinerea*. (B) Venn diagram of gene expression during the logarithmic and stationary phase of *B. cinerea*. (C) Volcano plot comparing gene expression between the two growth phases. (D) FPKM values of 10 selected genes corresponding to potential promoters during the two growth stages. (E) Relative expression levels of the 10 genes corresponding to the potential promoters during the two growth stages. Error bars represent standard deviation, n = 3.

To identify strong constitutive promoters in the genome of *B. cinerea*, a growth curve analysis was first performed. As shown in Fig. 1A, *B.* *cinerea* exhibited the highest growth rate at 24 h, and entered the stationary phase after 48 h. Therefore, *B. cinerea* samples cultured for 24 h and 96 h were selected for transcriptome sequencing. The analysis revealed that out of 12,576 genes with detectable FPKM values, 9994 genes were expressed during both the logarithmic growth phase and the stationary phase (Fig. 1B). Among them, the transcription levels of 10,602 genes showed no significant difference between the two growth stages (Fig. 1C). From these genes, 10 were further selected based on their high FPKM values, and their putative promoters were used for library construction (Fig. 1D). Detailed information on these genes is provided in Table 1. To further assess the transcription levels of these 10 genes at both stages, RT-qPCR was performed. As shown in Fig. 1E, the expression levels of all 10 genes were similar during both the logarithmic growth phase and the stationary phase, indicating that they are constitutive. In addition, we selected 1500 bp fragments upstream of the gene coding sequences as the promoter regions (Supplementary file: Table S3). The promoter region sequences of highly expressed genes were characterized. The results indicated that TATA boxes (TATAWAW), which are considered transcription start sites, were detected in the putative promoter sequences of Pthi4, Phs70, Paba1, Phy1, Phy2, and Pyqae. The TSS of the selected sequences were annotated, and the DNA double-strand stability profiles showed that approximately 1–4 TSS were detected in all promoter sequences (Supplementary file: Table S4).

**Table 1:** Detailed information of the 10 genes corresponding to stably high-expressing promoters in *B. cinerea*.

Gene	Enzyme function	Accession No.
thi4	Thiamine thiazole synthase	BCIN_07g06010
ef1a	translation elongation factor EF-1 subunit alpha	BCIN_09g05760
hs70	Heat shock 70 kDa protein	BCIN_03g06600
hs90	Heat shock protein 90 homolog	BCIN_10g00300
aba1	Cytochrome P450 monooxygenase aba1	BCIN_08g03850
hy1	hypothetical protein	BCIN_11g05380
hy2	hypothetical protein	BCIN_12g00640
fes1	Hsp70 nucleotide exchange factor FES1	BCIN_16g02010
yqae	Plasma membrane proteolipid 3	BCIN_14g04110
gdh	NADP-specific glutamate dehydrogenase	BCIN_16g04050

### 3.2 Measurement of promoter strength using the GUS reporter gene

**Fig. 2:**
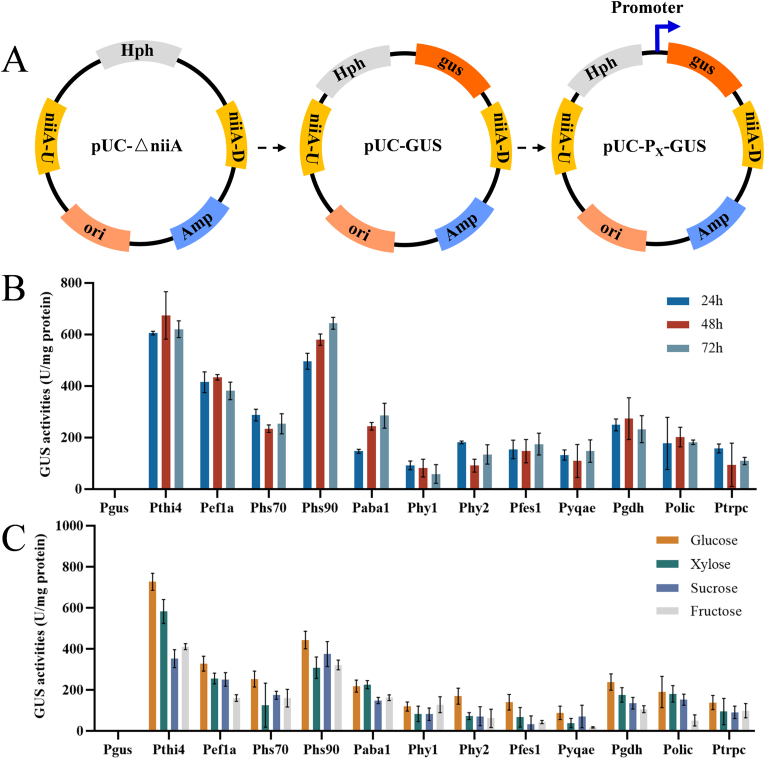
Measurement the strength of promoters using a GUS reporter gene. (A) Schematic diagram of plasmid construction. (B) GUS activity of *B. cinerea* recombinant strains with different promoters. The recombinant strain with the Gus expression cassette lacking a promoter fragment was used as the control group. (C) GUS activity of *B. cinerea* recombinant strains with different promoters in media containing different carbon sources. Error bars represent standard deviation, n = 3.

To confirm promoter strength at the protein level, GUS was selected as the reporter gene. In this study, a 1.5 kb fragment upstream of the coding sequences of the selected genes was amplified by PCR and used as the promoter region. It has been reported that knockout of the *niiA* gene does not affect the growth of *B. cinerea* in rich medium [24]. Therefore, this locus was chosen for the targeted integration of the GUS reporter cassettes. A series of GUS expression plasmids were constructed, as shown in Fig. 2A. Using the protoplast transformation method, the expression cassettes with GUS produced by the 10 candidate promoters were site-specifically integrated into the *B. cinerea* genome, and GUS enzyme activity was measured. Additionally, the *oliC* and *trpC* promoters, commonly used for heterologous gene expression in *B. cinerea*, were selected as reference groups, with a control group lacking a promoter.

Samples of *B. cinerea* were collected at 24, 48, and 72 h for GUS activity assays. As shown in Fig. 2B, the GUS enzyme activity produced by the *oliC* promoter was higher than that produced by the *trpC* promoter. Furthermore, compared to the *oliC* promoter, the GUS enzyme activity produced by the *Pthi4*, *Pef1a*, and *Phs90* promoters was relatively higher, with the *Pthi4* promoter showing the highest GUS activity, which was 3.34 times stronger than that of the *oliC* promoter at 48h. The GUS activity produced by the *Phs70*, *Paba1* and *Pgdh* promoters was similar to that of the *oliC* promoter. By contrast, the GUS enzyme activities produced by the *Phy1*, *Phy2*, *Pfes1*, and *Pyqae* promoters were lower than that of the *oliC* promoter. These results indicate that this study successfully generated a promoter library with GUS activity levels spanning 3 to 4 orders of magnitude.

To further investigate the expression conditions of these promoters, the engineered strains were cultured in media containing different carbon sources, and their GUS activities were measured. As shown in Fig. 2C, the promoters were able to drive gene expression in *B. cinerea* in media with glucose, xylose, sucrose, and fructose as the main carbon sources. Notably, almost all promoters exhibited significantly higher GUS enzyme activity in media where glucose was the primary carbon source, suggesting that glucose is the optimal carbon source for maximizing expression levels.

### 3.3 Applying the promoter library to tune the cofactor supply

**Fig. 3:**
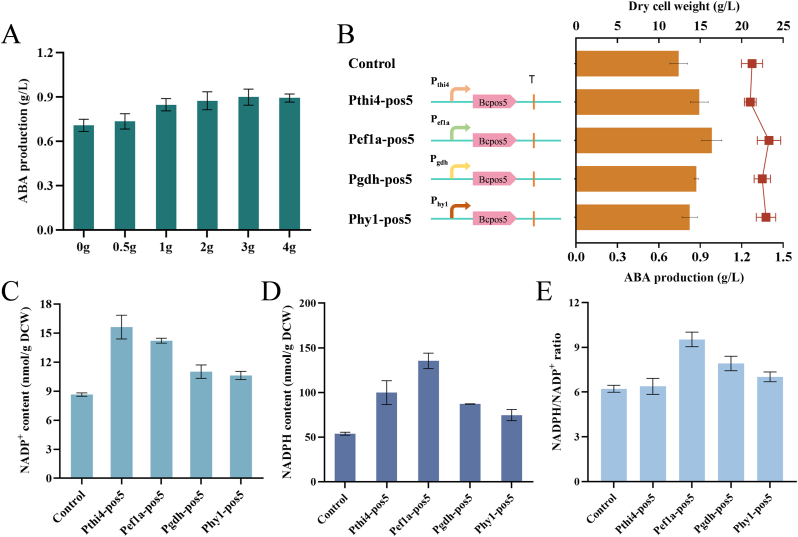
Applying the promoter library to tune the cofactor supply of *B. cinerea*. (A) The effect of exogenous addition of different concentrations of nicotinic acid ABA synthesis by *B. cinerea*. (B) The NADH kinase-coding gene *Bcpos5* was overexpressed in *B.cinerea* using promoters *Pthi4*, *Pef1a*, *Pgdh*, and *Phy1*, respectively. The effects of these overexpressed genes on cell growth and ABA biosynthesis were observed, with the line representing DCW and the bar chart representing ABA titer. (C) NADP^+^ content of the engineered strains Pthi4-pos5, Pef1a-pos5, Pgdh-pos5, and Phy1-pos5. (D) NADPH content of the engineered strains Pthi4-pos5, Pef1a-pos5, Pgdh-pos5, and Phy1-pos5. (E) NADPH/NADP^+^ ratio of the engineered strains Pthi4-pos5, Pef1a-pos5, Pgdh-pos5, and Phy1-pos5. Error bars represent standard deviation, n = 3.

The ABA biosynthesis pathway of *B. cinerea* has been elucidated in detail [25,26]. The gene cluster involved in ABA biosynthesis contains the four genes *Bcaba1*-*4*. Among them, *Bcaba1* and *Bcaba2* encode cytochrome P450 monooxygenases, and the oxidation reactions they catalyze require the cofactor NADPH as an electron donor. To investigate whether cofactor supply is a limiting factor in ABA biosynthesis, the effect of exogenous supplementation of the NADPH precursor nicotinic acid on ABA production was first explored. The results showed that the addition of nicotinic acid to the fermentation medium promoted the accumulation of ABA in a dose-dependent manner (Fig. 3A). When the nicotinic acid concentration reached 3 g/L, the ABA titer peaked at 0.9 g/L, representing a 27 % increase over the control. These results indicate that increasing the NADPH supply can enhance ABA biosynthesis, aligning with earlier research where the supplementation of nicotinic acid improved malic acid production in *Aspergillus niger* and d-pantothenic acid production in *E. coli* [27,28].

NADH kinase can catalyze the phosphorylation of NADH to produce NADPH, making it is an essential enzyme that regulates intracellular NADPH contents and NADPH-dependent biosynthetic pathways without directly affecting any metabolic pathways [29]. Extensive research has previously been conducted on NADH kinase in *E. coli*, *Yarrowia lipolytica*, and *A. niger* [[30], [31], [32]]. Additionally, maintaining intracellular metabolic balance is crucial for cofactor supply, and maximum expression of related genes is not always necessary. Therefore, this study aimed to tune the expression level of NADH kinase (encoded by *Bcpos5*) in *B. cinerea* using promoter engineering to enhance intracellular cofactor supply.

First, four promoters of varying strengths (*Pthi4*, *Pef1a*, *Pgdh*, and *Phy1*) were selected to regulate the expression of the *Bcpos5* gene. Plasmids containing the corresponding expression cassettes were then integrated into the *B. cinerea* genome via protoplast transformation, resulting in the recombinant strains Pthi4-pos5, Pef1a-pos5, Pgdh-pos5, and Phy1-pos5, respectively. Overexpression of the *Bcpos5* gene using these four promoters of different strength led to an increase of ABA production, albeit to varying degrees (Fig. 3B). Among them, the recombinant strain Pef1a-pos5, which used the *Pef1a* promoter, exhibited the most significant increase, with the ABA titer reaching 0.98 g/L, representing a 32.26 % improvement over the parental strain. The recombinant strain Pthi4-pos5, which used the strongest promoter *Pthi4*, exhibited the second highest ABA titer of 0.89 g/L, a 20.07 % increase over the parental strain. By contrast, the recombinant strains Pgdh-pos5 and Phy1-pos5, which used weaker promoters, showed small increases of ABA production. These results indicate that fine-tuning the NADPH regeneration system through promoter engineering can effectively enhance ABA biosynthesis.

To further investigate the impact of NAD kinase Bcpos5 on ABA production, the levels of NADP and NADPH were measured during the fermentation of *B. cinerea*. As shown in Fig. 3C and D, overexpression of NADH kinase using promoters of different strength significantly increased cytoplasmic NADP^+^ and NADPH levels. Compared to the parental strain, the NADP^+^ levels in strains Pthi4-pos5, Pef1a-pos5, Pgdh-pos5, and Phy1-pos5 increased by 80.5 %, 64.2 %, 27.3 %, and 22.7 %, respectively, while NADPH levels increased by 85.5 %, 151.6 %, 61.7 %, and 38.7 %, respectively. Notably, although the NADP^+^ content of Pthi4-pos5 was higher than in strains Pef1a-pos5, the ABA titer of Pthi4-pos5 was 9.2 % lower than that of Pef1a-pos5. We speculate that overexpression of *Bcpos5* using the strong promoter *Pthi4* in *B. cinerea* may have led to an excessive accumulation of NADP^+^ in the cytoplasm, which could have disrupted the redox balance, resulting in lower ABA accumulation. Additionally, we compared the NADPH/NADP^+^ ratios across all recombinant strains. As shown in Fig. 3E, Pef1a-pos5 exhibited the highest NADPH/NADP^+^ ratio of 9.52, significantly higher than that of the parental strain. These findings suggest that regulating the expression level of NADH kinase Bcpos5 in *B. cinerea* through promoter engineering can enhance the availability of the cofactor NADPH, maintain the redox balance between NADPH and NADP^+^, thereby supporting effective ABA production.

### 3.4 Combination of promoter engineering with a "push-pull" strategy to enhance ABA production

**Fig. 4:**
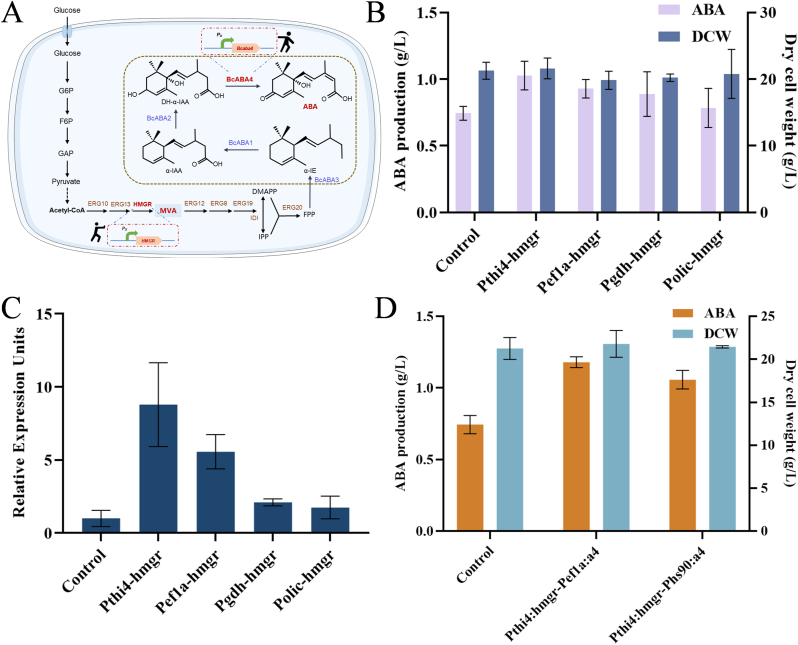
The enhancement of ABA biosynthesis capacity in *B. cinerea* through promoter engineering and a "push-pull" strategy. (A) Schematic diagram of the ABA biosynthesis pathway and metabolic engineering. The enzymes marked in red represent endogenous enzymes overexpressed in this study, and P_X_ represents different promoters used in this study for overexpression of target genes; (B) ABA production and cell dry weight of engineered strains overexpressing *HMGR* gene with promoters of different strengths; (C) Relative expression levels of *HMGR* gene, with the transcription level of the *HMGR* gene in the starting strain set to 1; (D) ABA production and cell dry weight of engineered strains Pthi4:hmgr-Pef1a:a4 and Pthi4:hmgr-Phs90:a4.

In *B. cinerea*, precursors for ABA synthesis are mainly provided by the glycolysis pathway and the mevalonate (MVA) pathway. Subsequently, farnesyl pyrophosphate (FPP) is cyclized into α-ionylideneethane (α-IE) under the catalysis of the sesquiterpene synthase BcABA3. Then, α-IE is oxidized to 1′,4′-trans-dihydroxy-α-ionylideneacetic acid (DH-α-IAA) by two cytochrome P450 monooxygenases, BcABA1 and BcABA2. Finally, DH-α-IAA is oxidized to produce ABA by the action of short-chain dehydrogenase BcABA4 (Fig. 4A). Among them, mevalonate is a crucial precursor for ABA synthesis, and enhancing its supply of mevalonate may have a positive impact on ABA production. Additionally, the *Bcaba4* gene plays a crucial role in the final critical step in the biosynthesis of ABA in *B. cinerea*. Research has shown that the expression level of the *Bcaba4* gene is closely associated with the ABA production rate of *B. cinerea* [33]. Therefore, this study combined promoter engineering with a "push-pull" strategy to optimize the biosynthesis of ABA in *B. cinerea* (Fig. 4A).

First, promoter engineering was utilized to investigate the impact of increasing the expression of the key enzyme 3-hydroxy-3-methyl glutaryl coenzyme A reductase (HMGR) in the MVA pathway on the biosynthesis of ABA in *B. cinerea*. Protoplast transformation was used to construct the four recombinant strains Pthi4-hmgr, Pef1a-hmgr, Pgdh-hmgr, and Polic-hmgr, each containing a *HMGR* gene expression cassette controlled by the promoters *Pthi4*, *Pef1a*, *Pgdh*, and *olic*, respectively. Fermentation experiments showed that the ABA production of the four engineered strains increased with the strength of the promoters (Fig. 4B). Among them, the engineered strain Pthi4-hmgr exhibited the most significant improvement, with a 38 % increase of the ABA titer, reaching 1.03 g/L. Further qRT-PCR analysis confirmed that the relative transcription levels of the *HMGR* gene were indeed elevated in all four recombinant strains (Fig. 4C). These results indicate that overexpressing the *HMGR* gene using strong promoters can significantly enhance ABA production. Additionally, compared to strain Polic-hmgr, which overexpressed HMGR under the commonly used *oliC* promoter, the strains that overexpressed *HMGR* using the promoters *Pthi4*, *Pef1a*, and *Pgdh* showed more significant increases of ABA production. This shows that identifying strong promoters suitable for *B. cinerea* can effectively enhance the biosynthesis of ABA.

Next, the "pull strategy" was implemented by overexpressing the *Bcaba4* gene to further optimize the ABA biosynthesis pathway. Since the engineered strain Pthi4-hmgr had the highest ABA production, we further used the *Pef1a* and *Phs90* promoters to overexpress the *Bcaba4* gene in strain Pthi4-hmgr, resulting in the engineered strains Pthi4:hmgr-Pef1a:a4 and Pthi4:hmgr-Phs90:a4, respectively. Fermentation experiments showed that ABA production significantly increased in both strains Pthi4:hmgr-Pef1a:a4 and Pthi4:hmgr-Phs90:a4, reaching 1.18 and 1.06 g/L, representing increases of 58.5 % and 42.1 % compared to the starting strain, respectively (Fig. 4D). These results indicate that combining promoter engineering with the "push-pull" strategy can effectively enhance ABA production. Furthermore, the cell growth levels of all engineered strains exhibited no notable variation compared to the control group, suggesting that alterations in the expression of critical genes within the ABA metabolic pathway did not noticeably impact cell growth.

## 4 Conclusions

This study established a library of native constitutive promoters from *B. cinerea* based on transcriptome analysis and promoter characterization. By using this promoter library to regulate the intracellular cofactor supply levels in *B. cinerea*, the biosynthesis capacity of ABA was optimized. Additionally, the biosynthetic pathway of ABA in *B. cinerea* was further optimized by combining promoter engineering with a push-pull strategy. As a result, the engineered strain Pthi4:hmgr-Pef1a:a4 with significantly increased ABA production was obtained, achieving an ABA titer of 1.18 g/L, greatly surpassing the titer of the parent strain.

To explore new strategies for optimizing the biosynthesis pathway of ABA in *B. cinerea*, we performed further analysis based on the transcriptomic data from Section 3.1. The comparative transcriptomic analysis revealed that most genes involved in the MVA pathway had downregulated expression, except for the genes encoding mevalonate kinase and phosphomevalonate kinase (Supplementary file: Table S5). Therefore, in future studies, increasing the copy number of genes related to the MVA pathway could be a potential strategy to optimize ABA production. Moreover, the expression levels of the key ABA biosynthesis genes *bcaba1-3* were significantly higher than those of most other genes, indicating their important role in ABA biosynthesis (Supplementary file: Table S5). However, there was considerable variation in the expression levels of the *bcaba1*-*3* genes. In samples from the two fermentation time points, *bcaba1* and *bcaba3* exhibited high expression levels, while *bcaba2* showed lower expression, suggesting that *bcaba2* might be a rate-limiting enzyme in the ABA biosynthesis pathway of this parent strain. This provides a potential target for future metabolic engineering efforts to enhance ABA production.

Additionally, we paid particular attention to the expression levels and variations of genes involved in the biosynthesis of secondary metabolites in *B. cinerea* (Supplementary file: Table S5). Four genes encoding sesquiterpene cyclases, three genes encoding diterpene cyclases, and nine genes encoding polyketide synthases were identified in the *B. cinerea* genome. The transcriptomic data showed that most of these genes, except for *Bcstc4*, *Bcphs1*, and *Bcpks4*, were not expressed. This suggests that, during the fermentation process for ABA production, the biosynthesis genes for various secondary metabolites remain silent. Therefore, in future studies, knocking out *Bcstc4*, *Bcphs1*, and *Bcpks4* may increase the carbon flux towards the ABA biosynthesis pathway, while other secondary metabolite biosynthesis genes could serve as potential integration sites for metabolic engineering in *B. cinerea*.

In the future, with further optimization through systems metabolic engineering strategies, the production level of ABA is expected to be significantly enhanced. This study provides important guidance and references for enhancing the biosynthetic capacity of *Botrytis cinerea* and promoting its large-scale production of ABA.

## CRediT authorship contribution statement

**Ling-Ru Wang:** Writing – original draft, Investigation, Data curation. **Ji-Zi-Hao Tang:** Investigation, Formal analysis, Data curation. **Shu-Ting Zhu:** Writing – original draft, Data curation, Conceptualization. **Na Wu:** Resources, Methodology, Conceptualization. **Zhi-Kui Nie:** Writing – review & editing, Supervision, Methodology. **Tian-Qiong Shi:** Writing – original draft, Supervision, Funding acquisition, Conceptualization.

## Declaration of interest statement

The authors declare that they have no competing financial interests.
