# Supplementary material for: Enhancing abscisic acid production in Botrytis cinerea through metabolic engineering based on a constitutive promoter library
Source: Synth Syst Biotechnol. 2024 Dec 20;10(2):373–80. doi: 10.1016/j.synbio.2024.12.004 (PMC11742572; doi:10.1016/j.synbio.2024.12.004)
Supplement: Multimedia component 1 [file mmc1.docx]

**Supporting Information for**

**Enhancing abscisic acid production in *Botrytis cinerea* through metabolic engineering based on a constitutive promoter library**

Ling-Ru Wang^a^, Ji-Zi-Hao Tang^a^, Shu-Ting Zhu^a^, Na Wu^b^, Zhi-Kui Nie^a^, Tian-Qiong Shi ^a^*

^a^School of Food Science and Pharmaceutical Engineering, Nanjing Normal University, Nanjing, 210023, PR China

^b^College of Marine and Bioengineering, Yancheng Institute of Technology, Yancheng, China

*Corresponding Author: Tian-Qiong Shi

Address: School of Food Science and Pharmaceutical Engineering, Nanjing Normal University, Nanjing 210023, P.R. China

Email: [tqshi@njnu.edu.cn](mailto:tqshi@njnu.edu.cn)

Table S1 Strains or Plasmids used in this work.

| Strains or Plasmids | Characteristics | References |
| --- | --- | --- |
| *B. cinerea* NNU-1 | Wild‑type | Our lab |
| *E. coli* DH5α | Wild‑type | Our lab |
| Plasmids |  |  |
| pUC57 | Amp | Our lab |
| pUC-△niiA | pUC57 derived, up and downstream fragments of niiA, a hygromycin expression cassette | This study |
| pUC-GUS | pUC-△niiA derived, GUS coding sequence | This study |
| pUC-Pthi4-GUS | pUC-GUS derived, promoter Pthi4 | This study |
| pUC-Pef1a-GUS | pUC-GUS derived, promoter Pef1a | This study |
| pUC-Phs70-GUS | pUC-GUS derived, promoter Phs70 | This study |
| pUC-Phs90-GUS | pUC-GUS derived, promoter Phs90 | This study |
| pUC-Paba1-GUS | pUC-GUS derived, promoter Paba1 | This study |
| pUC-Phy1-GUS | pUC-GUS derived, promoter Phy1 | This study |
| pUC-Phy2-GUS | pUC-GUS derived, promoter Phy2 | This study |
| pUC-Pfes1-GUS | pUC-GUS derived, promoter Pfes1 | This study |
| pUC-Pyqae-GUS | pUC-GUS derived, promoter Pyqae | This study |
| pUC-Pgdh-GUS | pUC-GUS derived, promoter Pgdh | This study |
| pUC-Polic-GUS | pUC-GUS derived, promoter Polic | This study |
| pUC-Ptrpc-GUS | pUC-GUS derived, promoter Ptrpc | This study |
| pUC-Bcpos5 | pUC-△niiA derived, Bcpos5 coding sequence | This study |
| pUC-Pthi4-Bcpos5 | pUC-Bcpos5 derived, promoter Pthi4 | This study |
| pUC-Pef1a-Bcpos5 | pUC-Bcpos5derived, promoter Pef1a | This study |
| pUC-Pgdh-Bcpos5 | pUC-Bcpos5 derived, promoter Pgdh | This study |
| pUC-Phy1-Bcpos5 | pUC-Bcpos5 derived, promoter Phy1 | This study |
| pUC-HMGR | pUC-△niiA derived, HMGR coding sequence | This study |
| pUC-Pthi4-HMGR | pUC- HMGR derived, promoter Pthi4 | This study |
| pUC-Pef1a-HMGR | pUC- HMGR derived, promoter Pef1a | This study |
| pUC-Pgdh-HMGR | pUC- HMGR derived, promoter Pgdh | This study |
| pUC-Polic-HMGR | pUC- HMGR derived, promoter olic | This study |
| pUC-△niaD | pUC57 derived, up and downstream fragments of niaD, a G418 resistance expression cassette | This study |
| pUC-Bcaba4 | pUC-△niaD derived, Bcaba4 coding sequence | This study |
| pUC-Pef1a-Bcaba4 | pUC- Bcaba4 derived, promoter Pef1a | This study |
| pUC-Phs90-Bcaba4 | pUC- Bcaba4 derived, promoter Phs90 | This study |

Table S2 Primers used in this work. All primers are synthesized by Synbio-tech Co., Ltd., China.

| Primers | Sequence (5´-3´) |
| --- | --- |
| BcniiA-U-F | gctcggtacctcgcgaatgcatTGTGCTACTTCATTGGAGAAA |
| BcniiA-U-R | GACCATCTCTGTCCGCAAAGA |
| Polic(niiA)-F | TCTTTGCGGACAGAGATGGTCGCctgcagctgtggagccgcat |
| olic-R | ggatcgattgtgatgtgatg |
| HPH-F | catcacatcacaatcgatccATGAAAAAGCCTGAACTCAC |
| HPH-R | CTATTCCTTTGCCCTCGGAC |
| Ttrpc-F | GTCCGAGGGCAAAGGAATAGCACTTAACGTTACTGAAATC |
| Ttrpc-R | GATTTCGAGGTTTATACCTA |
| BcniiA-D-F | TAGGTATAAACCTCGAAATCgcggccgctgccacctagtcaattcatc |
| BcniiA-D-R | ggcccgggatccgatatctagCAGCAGGTATCGACCCAGAAG |
| GUS-F | AACCTCGAAATCgcGGCCGCatgttacgtcctgtagaaac |
| GUS-R | tcattgtttgcctccctgct |
| Tgluc-F | agcagggaggcaaacaatgacgtatgtagataagatgtat |
| Tgluc-R | TTGACTAGGTGGCAGCGGCCatcttgttggggggaagggg |
| Pthi4-F | GTATAAACCTCGAAATCgcGGCCAACAAAAGGCCTTCATTTGAGA |
| Pthi4-R | ctacaggacgtaacatGCGGCCTTTCGATATTTTTCAAGTTGTT |
| Pef1a-F | GTATAAACCTCGAAATCgcGGCCACACACACCACTGAAGAGTGAA |
| Pef1a-R | ctacaggacgtaacatGCGGCCAAGAAGAAACAAATCAGTAAGA |
| Phs70-F | GTATAAACCTCGAAATCgcggccCCTCCTCACCTTGACATTAATC |
| Phs70-R | ctacaggacgtaacatGCGGCCGTTAGTACATGTGTCTGTTGTG |
| Phs90-F | GTATAAACCTCGAAATCgcGGCCCAGGCATATTCTGGAATGCATC |
| Phs90-R | ctacaggacgtaacatGCGGCCATTTGTGATTGTGTATGTAAAA |
| Paba1-F | GTATAAACCTCGAAATCgcGGCCTTATAATCTATAAATACTAAAT |
| Paba1-R | ctacaggacgtaacatGCGGCCGGCTTTTGCTTTCGTAGGAAGG |
| Phy1-F | GTATAAACCTCGAAATCgcGGCCTTTTGCAAGTATTCAATTCCGC |
| Phy1-R | ctacaggacgtaacatGCGGCCTTTGATTTTTGGGGAGGAGCGA |
| Phy2-F | GTATAAACCTCGAAATCgcGGCCTCAATGAGATTACCATCTGGGT |
| Phy2-R | ctacaggacgtaacatGCGGCCTGTGGTTGAATTGATTTACTTG |
| Pfes1-F | GTATAAACCTCGAAATCgcGGCCTTATGAAGGGATTGCTGCAGGC |
| Pfes1-R | ctacaggacgtaacatGCGGCCGATGGCAGATTTCTATGTTCTC |
| Pyqae-F | GTATAAACCTCGAAATCgcGGCCTCGATGGTCAATACTTTTTGAT |
| Pyqae-R | ctacaggacgtaacatGCGGCCGATGTTGGATTTAATTGGTGAA |
| Pgdh-F | GTATAAACCTCGAAATCgcGGCCATTAACCACGCATCTCCTCATC |
| Pgdh-R | ctacaggacgtaacatGCGGCCGATTATTATTGTGGGTGTTTGG |
| Polic-F | TAAACCTCGAAATCgcGGCCctgcagctgtggagccgcat |
| Polic-R | acaggacgtaacatGCGGCCggatcgattgtgatgtgatg |
| Ptrpc-F | TATAAACCTCGAAATCgcGGCCTCCCAATACAAGTGTAATGCTA |
| Ptrpc-R | ctacaggacgtaacatGCGGCCTTGGATGCTTGGGTAGAATAGG |
| Bcpos5-F | AACCTCGAAATCgcGGCCGCATGTTTCCGAGGGTGTTGCC |
| Bcpos5-R | atacatcttatctacatacgTTATTCTTCGCCAAAAGGAT |
| Tgluc-2F | cgtatgtagataagatgtat |
| Pthi4-2R | ACCCTCGGAAACATGCGGCCTTTCGATATTTTTCAAGTTG |
| Pef1a-2R | ACCCTCGGAAACATGCGGCCAAGAAGAAACAAATCAGTAA |
| Pgdh-2R | ACCCTCGGAAACATGCGGCCGATTATTATTGTGGGTGTTT |
| Phy1-2R | ACCCTCGGAAACATGCGGCCTTTGATTTTTGGGGAGGAGC |
| HMGR-F | AACCTCGAAATCgcGGCCGCatgattggaaatacgctcct |
| HMGR-R | atacatcttatctacatacgctatttctttccccctgcag |
| Pthi4-3R | gtatttccaatcatGCGGCCTTTCGATATTTTTCAAGTTG |
| Pef1a-3R | gtatttccaatcatGCGGCCAAGAAGAAACAAATCAGTAA |
| Pgdh-3R | gtatttccaatcatGCGGCCGATTATTATTGTGGGTGTTT |
| Polic-2R | gtatttccaatcatGCGGCCggatcgattgtgatgtgatg |
| BcniaD-U-F | gctcggtacctcgcgaatgcatTTCTACAACCCCACAATCCCG |
| BcniaD-U-R | CAGGGAAGCCATCATACGATC |
| Ptrpc-2F | GATCGTATGATGGCTTCCCTGGCGGCCgctcccaatacaagtgtaatgc |
| Ptrpc-2R | TTGGATGCTTGGGTAGAATAGG |
| G418-F | CCTATTCTACCCAAGCATCCAAATGATTGAACAAGATGGATTGCA |
| G418-R | AGTTATCTCGAGCGATACGCTCAGAAGAACTCGTCAAGAA |
| BcniaD-D-F | GCGTATCGCTCGAGATAACT |
| BcniaD-D-R | gggcccgggatccgatatctagAAGCAGACTGCGTTATTGTTT |
| Bcaba4-F | TGATGGCTTCCCTGGCGGCCGCatgtcctctcaaccattcacg |
| Bcaba4-R | ctaacatctccatccgccat |
| Ttrpc-2F | atggcggatggagatgttagCACTTAACGTTACTGAAATC |
| Ttrpc-2R | CACTTGTATTGGGAgcGGCCGATTTCGAGGTTTATACCTA |
|  |  |
| Pef1a-4F | TGATGGCTTCCCTGGCGGCCACACACACCACTGAAGAGTG |
| Pef1a-4R | ggttgagaggacatGCGGCCAAGAAGAAACAAATCAGTAA |
| Phs90-2F | TGATGGCTTCCCTGGCGGCCCAGGCATATTCTGGAATGCA |
| Phs90-2R | ggttgagaggacatGCGGCCATTTGTGATTGTGTATGTAA |
| Qthi4-F | GATGCCTTCTTGACTGAT |
| Qthi4-R | AGAGTTGATGTGAATAAAGC |
| Qef1a-F | AACTCATTGTTGCCATCA |
| Qef1a-R | TTGTATCCGACCTTCTTG |
| Qhs70-F | GTCCACGAGATTGTCCTT |
| Qhs70-R | GTTGAAGTAATCGGTGATGAG |
| Qhs90-F | AAGATTGAAGAGGAAGAG |
| Qhs90-R | TGTAGAATGAACCGTATT |
| Qaba1-F | CTTCGCTTGCTTGCTGAG |
| Qaba1-R | TAGAGGATACCACGCTAAGAC |
| Qhy1-F | GAGACCTGTTAGTAGAAC |
| Qhy1-R | AATACCTCCTCCTATAAGT |
| Qhy2-F | AGGATATGAAGAGGATAGAC |
| Qhy2-R | GAATGCCTTGAATGACTT |
| Qfes1-F | ATACGCCGTTCAATCTAC |
| Qfes1-R | CGAGGTTCTCAATCAGTT |
| Qyqae-F | TTGGTGTATTCCTTGAGA |
| Qyqae-R | TAATGTATAGAGCGTGGAT |
| Qgdh-F | CCTACCGTTAATCTCAGTGTT |
| Qgdh-R | TCCAATGTTCAATCCAGTCA |
| Qtub-F | TGAAGGTATGGACGAGAT |
| Qtub-R | GCATCCTGGTATTGTTGA |
| Qhmgr-F | TACAAGCACTACAACTATGA |
| Qhmgr-R | GAGAGGCAATGGAAGATA |

Table S3 Sequences of the selected promoters of *B. cinerea.*

| Promoter name | Sequences |
| --- | --- |
| Pthi4 | AACAAAAGGCCTTCATTTGAGATTGACTAGAGTATTAGGGTGGCTCCTTCCTAGTACTAGGTCTGCACAGAAAGAAGAAGTCGGGAATGTGTAATTGAGCTTTGACTGAGCTCTGCATCTTCAGGAATTATCCTACTATACCGAAGTTGTTTGTGAAAATAACCTATGCTAAGCGTTTCGCTCATACACAGCTGGAGTGCACATCGGAGTATAGTGCAAGGAAGGTTCTTCATGATACTGTTATGACCTATTCCAAAAACAGAAATTACTACAGTAGATATTATCTGTCCCTGTGTTTCTTAGCAATTGCTTCATTGCATTTTCTTTGCTTCATGTGATCCATATCTAAGAATATTTGCATACATATGAAGAATTCAAAGAAGCTTGTATTGGTGTTATAGTTGAGATTCTCGTGAGAATCATTGGAATCTACGAAGCATGAATGGCTGGCCATGATAGGAACTAGAGTGCCAATTGATGACTGGGTCTTTCTTGAAATTTGAGAGTTGAGGTATTTCTTATTCTGGCTGAGGGACAGAGAATTGCCAGGCTTACTCAAGCGGAATTTAATGGAACCAAGCATTGCTGATTGCTGCTAGATGTATACAAACCCAGCCCAGCCATTTTTCTCTCTCAATCGAACGCCGCAGCTGTGAGTCATTGTCCTGGAAAGCTTTTTCAAACCACAAAAGCAGGTAGACATGGGTTTTGGTATTGCCTTTACCCATCTCTCCGTGATTCGGTGGATGTTGATATATTCCAAGTCTCGCAAACCACTGCAGGAAGCTATGAGCTCACTCCAATCAAAGTGTGCCAGTTACATACTATGACAATTAGGATCGGTGATCAAAATTTGACAGTCCAGTGAGGTAAAACTTTGACATCCCAGGTTTTGGATCGGTCAATCAGATGGCAGCGATAGCGATGTTGGTGCACCTTAAAGAGGTTAGGTTCTGGGCACGGGGCCACTAAAAGTTTACCCCTCACTTAGCTCATGATTTTCGGACTACTTAATCTCTGTATGATTCCCAACACAATCTGTTCTTTTCCCTCCATCTTCCAGAAAGCTCGTCACTTACGTATCAAAACTAAACAAACGTTCATGTCAGTCATCTTGCACCAATTGGAGGGAAATACTGATGTTGTCTACTCATCCTAGCAAACGACTACTCGTACCAAGTACAACTCACTTCCAACAATTGGAAGTCATGTAATCCTCAGCATGGACATTATCCGCTTCGCGGATCAAGGTATCGCATCAATTGTATAAGTCACTCTCGCATGAACCGGTGTTCGATCCTGCAAATCGTCAGCCTGCCTCTCAATTACCACCTTACTCAGTAAGATGATATGAGAGTGCCGCCGATGAGGGGTCGTTCTGAGATTATACGGTTATATGAACTTGATCTGGATAATACCAGCGAAAGGATCATGCTCTTCCATTCCGTCCTTATGGACGTCACTAATCTCTCGCAGGTAACAACTTGAAAAATATCGAAA |
| Pef1a | ACACACACCACTGAAGAGTGAATAAGCTTACCGTCGACTTGACTGCGTTTGGATGACACTCGAATTCCAAAAACGATCCCGACTGTTCATCTCAAGCCAGATGAAACATCCATAACCGCATCAAGTATGGAATATGTACTTCCATTCAATAATCTGACGCCAGCCTCGTTGTGCAGGAAATGTCAAGAGATATAAAACTGATTGGCGATAAGTGGGGGGTGAATGAATACTTTTTTTTTCTTTTCTTTCGAAACTCGCATCATAATCTTGGGTTTCAATTCCCATTTATCCACTGATATTCGTTACTTCTCCTCACGTACCATGAAAATTTGCAAGTGAATGGATGCAGGTGATGCAACGAGAACCCACTTGTCGTGAGAACACGGTGACACTTCATCAAAATTGATCAAATCTTGAAGCCATGGAGGCCGTTCTTGCACCCAGTTGCATGATATCAGAATTGCCCCTCACTAAATTTGCTTGCCCCTCCACCAAAGATCCCAAAATGAATGAAAATTTGAGTCAAGGATTCTATCAAGATAGGAATTCACTAACCGCTTGTACAGATGGTTGCCGCTGGGGAATCCACATTGATTTCATCGATTCCACTTGGGTGCATAAAGGAGCAAAATTGAATGGGAGTGATTGGCTTTCGTATAATCAAGATAAAACCTAATGTACGATGTCCTCGAGCAACGAAATAGTCTTGTAACTTGTGAGCCTGAGCCATCCTGGGAGCCCAATGGTCCCAATCGCCACATTTATCTCATTGTCTTCTTTTTCCCGGGCAAAACCTTCATTTTGTGGCCCATTTTTGAACCATTTTTCACTTTTTTTTTTTTCGCTTTTTCTCTGCTGGGAACCTTTCCCTTAGACGTTCAAGGTTGTCTTATTTTGTTTTGGGGATTTTCATGAGTTATGACCACAAAATAGCTAGTCTCAGCTAGTCCACCATACAGCAGCTAGCCATACGCTAGATGTGAAAGAAAAGAGTGAATGTAGCAAATGAGAATGAATCCACTTCCAATATTCACCGGACCAGTCAGACCGTCGTAAATGACTGGAAAGTAAGCGTTTTGGTTGGAGTCACATGATATACCCCACCAACTAGCGTTCAAATTCTATCGCTTAGCGTACCGCACACATCAACTTTTTTTTTTATAAACCCCGCCATCCTCTCGAAACTCTTCTTCTTCCTTCTTCGTCGCACATACCTGGATTCAAGTAATCATACCTGTGCAGTAAGTTTAAATTCCATCCATTCTTCCATCCATCGGTCTCTTCCTCTTCTTCTTTTTCTTCTTCATCAACCTTCTTGAAGATCGATCAACTAATATCATCTTCTTTCGATAGATCTATTTACGAAGACAAAACTTCCACAAATAAACAAACACAACCATCAAAATGGGGTAAGTCTCGAAGTCCATCAGAGTTTTCCATCAGCATTATCTCTCACCCATCGATCTACATCGTGGACTTCTTACTGATTTGTTTCTTCTT |
| Phs70 | ACTACTACTACTACTACTACTACTACTACTACTACTACTACTACTACTACTACTACTACTACTACTACTACTACTACTACTACTACTACTACTACTACTACTACTACTACTACTACTACTACTACTACTACTACTACTACTACTACTACTACTACTACTACTACTACTACTACTACTACTTAATACTACTCCTCCTCCTACCTCCTCACCTTGACATTAATCAAACTCCTGCATCTTGGTTGCCCTTCACAACCATATAATGACACCATTTTTCATTATCATTATTGACTTGCCCGTGCCAAAAGCCCCCATCATTTAATTGTGACGTGATAAGGACCTGGTGGGGCTGAGGGGCCTTTGCCTTTCTCTGCTCTGTAAGTAATTGTAATTCCGACAACCGCTGTGAATTAAATTCTCCAGAGGACGATGAGACGGGAAAAAGGTACGCTTGCTCTATACATGTACTCGATACTCGATCCTCTATCCTCTATACTCTATGCTCTATGCTCTATACTCTATACATGTACTCTACCCTCTATCCAATCAATCTTGCTTCTGCGTCGAGTGGAGCACGAATGAATACGACCAACGATGTGATATCATGTCCCTATCAAGTGCAGAGAGTTACTAAAAGCCAAAGCGATCAAGATTTCATCCGATTCTTGAACTTTCTTTCCCTTCTTCCCGTGCAAATTACAGTGGAGTTCATGTCATAAATAAATCTGGGCAAGGGAAGTTTCTTTGACGGGGCGACGTCATGGTCATGGTCATGGTCATGGTCATTGGACGGGAAGGCGGTAGAAAACGGTAGAAACAGAAGTATGAGGCATGAGGCTATGGTCTGTGTGGGCTGGCTGTCATAGGTCCTTATGTAATCCACGTCGATTGAGGGGCAATCGGGCAAAAAAAATAAAAAAAATAGAGTCATCAAAGAAAGGGAATGGCATCCAAATATCTTCACCATGTCCATCAACAACACCATGCATGTAGAATAGATCCGTACACATCCAAGTGATATCATGCCGTTCCATCGACATCCACCGTGTATCTCCAATGTAGTGAAAAATCAAAAATCAAAAATCCATCCACATCTAGACGATTCTTTCCATCGCGATTACGAGGTCGAAGCTGATGGTGATTTCGAGAGCTTCCCACCGCCTTCTCCTTACGTCCATATCCACCGCTTTAAGGCAAAGAAGATTCTGGAATCGAAGCTGCTGGAATCGAACTTTTCGCTTTAAAAGTCTCACAGTCAACCCAACTTTTTCCTTCTGCATATTTCTCAAACAATTCATTTCAAGATACCACTTTTGCAACGACTCTTCTCTATTTTCATCCAAAAAGCGTTCAAAGAAGTTACGATACTTCGTCTTTCAAATCCATATACACACAACTAGATTAATACAAGATGGCTCCAGCGTAAGTGACTGACTCCATTTCCTTCCGTCAACCCCTCCTTTCAGCCTTGCAACGTCAATCACAACAGACACATGTACTAAC |
| Phs90 | CAGGCATATTCTGGAATGCATCACAGAAATGGAAAAATTGGTAGGTCTAGATTTTGGTTGAGTCACCGGCCACTAACCAGGCACTCCATTCCCTCGGGAAAAATCTACTGCTGAATTCCTTTGGGGGGCGATGACTGGGGATGATCAAATTGGGAACTTGAGAACACGATCCCGTTGAATTTTGTGTGCGTGTATAATCTAGATGTCAAGTTGTAAGAGAGAATAGTGCAATAGCCACCTACACTATGTGCCATTAGATTGTCAATATGATATGATATAATGCGGAAAGAAAGAACCCAAGCTGCCAAGGCCTACTATTAACTTGCCTTCGATAAGGCTGGCAAGCTGGATTCTTTTCCGTGGCTGTCAAGTCTATTCATGGCCTAGAAGGCTTGGGGGGTGAGAAAAAAGGTCCAACATTTTTGTCACCGCCACTGCACTGACAAATTCAAGGGGTAATCGAAGCGTCTCGAATGGAAATTTCAATGTAGTGATGATATGATCGTTAACAGTCATAATAGGAATCGAAATCAAAGGAATCTTCAAGGACTCCCTCAAACTTACTAGAAGCTTCCGAGGCACGTGAGATTACCCACACGAGCCTCATTCACCAGCCTCAATTGTGAAGCACAGAACAGAAACCGGTCAAAGTTTTTCCCCGCTGATAAGATGAGCCACTGCGATAAAGAAAATTGAGATTCTAGAGGGGGGAGAAGAAGAAGAAGAATTGATTGAGATGAGAAAGAAGAGATGGCACCGAACAATTCAAAGAGACGAGTAATAAATGCAAAGTCATGTGAATTCATCTCTCTTCATAGGATGTATTGAGAGTACCAAGATTACTAAGCAATACCCTGGCCGTCATAGATATGTTTTGACCATGTATTGACTGTTTGCATTCAATTCCCCCAAGTGCCCACAGCTACCTAGCTATCCATAACTATACGAGTAATTCTAAAAAGTTCCATATACATCACATCGTTTGTCTTGCCTTGTGCCTTGTCATGTTTTCTCGCCCATTGATTGGCTGGGCCAGAAGTGGCGCGTTGCTAGGTGGTCTAGAAAGGGTTAAGCGACCAGAAAGTTCCTTACAATTCTACCATTTTCTATAGCGAGCCTTTCCATAAAATCCCCAATACCCTTCAACGAAAAGCCAATTCTCTTTTTTTCTTCAAACCATTCAAAGATACCCTTCCTTTCTACTTTCGGTCTATTAGACTTCTACAATACGATACACTTAACGATTTAACGAATACCATCAACTGTAAGTTACCTTCAACACTCTACCTGTGTCGAAGCAACCCCTCGATCTCCCATCAAGAAGCCCTTTGGAGAGCTCAGCCTCGAAAGAGAAAGAGATCCTAGAAAGCGTTACTTGTATCCTGCAAGAGATTCACAACAGGCTCAATAAGCTTGCACAACCTCATTTCCCATCAAGAAATCAATACTAATCTATCATCTTCCTACAGTTCCTCCGTTTTTACATACACAATCACAAAT |
| Paba1 | TTATAATCTATAAATACTAAATCATTAATGACTAACGTAGTTTATCATCGAGTGGGCCACTATAAATACATACAAAATTCGTCTGTGCGTAACCGTGCACTTTCTTCATTTAATAATATAATTTTATAAAACTTGAAATTATGAGATCTAATTATATAAATATTATAAAAATCTAATTTGTTTTTCAAAATTCTAATTAATACAGGAGTTATTATTGAAATAATACTTTTGTAACGAAAATCGTGACAATTTCTGATTTTTATAGAATATAATACAAATTTTCATATAAAAATTAAATATTTGTATAAGAAATCTTTTCTTATTGATAAATCTTTTTTCTTTTCCAAAAATATTGGTTAATTATTAAAATGTAAATATTATGAAAAGGCTAATTTTGGTGTATTTTGAAGTGGCCCACTCGATGATATGGCCCACTCGATGATAAACTACGTTATTCTAATATAAATAATATAATAGATGAAAAAGAAAAAATTATTGGGTGAAAAGGAAATTGACTAATATGTTCCAGATTATTTTCAAAGTTGAATAATGATATATTAGGCGAAAGAAATCATTCTGAGCAAAAATGTCCTACGACGCGTCATACCTTCCTTACCGCCAGATATGAGTCAACATCAGTACAATATAATGTAGAATCGCTTTTATAGTATAGCTACGCAAACGTGCCCAATCACTGTGATTATCACAATAGAGGAAGCCCTTGGAGAAATGTGTAACTCCCCTTGCATGTGTTAAATCAACTGATCTGGCCCAAACACGATCGTGTCGCAAGCGTTCCGATTAGTGCCACTTTTTGTGTTTTTCCCTGTACTCAGTAACACAAGAGTATCTTTAGTTTCATTTGTAACTTCCCTAGGTTTTCCATTGGAAACTGGAGAACAGTAGTCTTGTCTCACATAATGTGGGCTAACTACTCTGATAATTAACTCCGTCAGCTGCATTGCTTATGTGCATGAACGTTAAATTTGATATGGTTCATAGGAAATTTTCGCCCTTTAAAGCCATCTTGTATTCGTCACCACCTGAAGTTTGCTGCCCCGATTTCAAATTAGACTTTCGGGTGTAACCGTCCAGAAAGCTTAAAATTTAGCCGAACTACCTTCCATCTTCAGAATAGGAAATTGACAGGTCCAGTTAATAATTCGCTATGATAGGTGTTCTTGTGGAAGTCATTGGAGCCAGACTCTCATTTGACATGTGGACATCATGCACACTTATCACCACTCGCGGATCTGCGTTCTCGAGAGATCTTCGTACTCTTAGCCACATCGAACTACGTATTATATTTTCTAGTTTAAGATTGCGTGGTATAAGATATTACTGCCTGATTCTCTGCGATAACACAATCCTTTCTTCACTTTTAACATCCATTACAATACTCTCCTGAACATTTTCCTGCATGGTCTACTTGATAAAAATTTGAGATCTTTGTATCATTCAACATCGTGGACGACTGGCCTTCCTACGAAAGCAAAAGCC |
| Phy1 | TTTTGCAAGTATTCAATTCCGCAGCGCAATACCCTCCTCCCTCCATCCTTACCCCGCAAGAATGAAAAAATAAATAAAAAAATCAGACGGTGAAGAGGAAATTCTAATCTTAGGGATGTTGGTTGTAATGTTATCCTAGCACGCCCAGCTAGAGAGTACGCAAACGAAAAAAAACTAATGCAGTCTGACGTCGGTCCCTTAAAAGTTAATTGATCTACCAACTGACTGATAATAACAAGGTCTAGTAGGTAGCAAAGGCTTTGAAAATCCCTTTGTACGCTGGTTGAAATTGAATCTAAGCTGGTATTTATTCTCCATTCGCCCTTCTCCATTTTTGATGGACGATTGACGATTGATGGTTGCCGATTGATGATCTGTGCTGTGAGCAAGTGCTTCGTAGGTGGTGATCATATCGCGATCAACAAGGCATGTAGGTACTTAGAGATGTGAATAACAATTGAAGAAAGAGAAAGATAGATACATAGATGCATCTGGATCCGATATAAAGTTCGACCTTGCCTACATCTGGATTGTCAGACTAGATGCATATACACACTGCCTAGCTATCTAGATACATATTTTCTCATTCGTTCATCCATGGTTTACGAGGTTTCGGGGAGGATTCTTGCTTCTTGGGGCATGGTTCAATGGCTAGGAGGGAGAATGGTCGAGATGGATCGGTGGATCGTGTGGTGGATGTTGATGTGTGTTGTGCTTGTGAGGGAGGGTCGGTAGATGAGTAGTTTGAAATGTATCTCGAACGCCATCCAATCCCATCCATCCCATCCATCCAATCCAAGTCCAAGAAACACAGATGAACTCGATAAACTTTCCCGCATCACTCACGACAGCCAATATGGTGGAGGCTGGGCTGGTGGGTGGGCTCTTAGCGATTTCTAGATTTCCTAGTCCCGGGGTCGATGTGGTGGAGTCATGGTGGTGGATACCGGTGCGCACGATACATGGAGTCTCTTATCAGAACGACACAATGTATATAAGAACCTGGGTATTGCATAGGAGAACGATCTAGCTGGGTGTGGATTCATGGGGGATCGTGGTACGATTGTTATGCAGTGTGGACTTGGCTGATGTGATGGAGTGGCTAGGTATGTTGTATGACATCTGAGGGGTTTAGTCAATTACTTGATTGGAGATGAAGTAAGTGGATGGATGGATGAGATGGGAGGTTTATAAATGTGTGTTTTTCCTGTGTTGATGTGATTTTGTAGTCTTGCTGTTATTTCACTTTGTCGAATTTATCCATATATAGAAATCCTCATCTTCCCACTCGGTAAATTATTACTCAACGAACGAAACTAAAAAAGAAAGATACGCATTCGATTTCGATATCGGGATTCGAGAATCAATACAATACAATACAAAACTTCCACCACGATTATTATCACACACATACATCTACTCACTCTTCCTCCAAACCAACACACCAGCACACAGACAATCGTACCTCCCTACAAACCTCGCTCCTCCCCAAAAATCAAA |
| Phy2 | TCAATGAGATTACCATCTGGGTAGTAATATTATTACGAGCAAAATTGCAATGCAAAGAAACATTGGCCGCGTAACTCTATATCTATTACCTCGAATAATCAAAATGGTGTTCGACGATCGACTACCCGACGATTGTCGAATATCATAGTGGCTCGAGTGGATAACTTTGCTGCCAAGTAAGTATAATACCTCTTGTTATACCTCGTGACCACTTGCACGATTTCCACAGAAGAAATTTCGGACTCCCCGATCTAGCAAGTATCTCCTCAGTACTGAATCGGCACCGAACGGGGAAACCTGTGTGTATTGCGAAACCACACTCAGACACCCCACTGAATCAGAACAATACACAATGTCAGATACCATCAATCCGGCCCCTCCCATGTTTTCCCTATCATGGGCGAAAATGATCACGAGGCTTGAAAGTGGAACACACTGCATAGACAACACATGTGTTCATTTGTTCATACAGCAACTATTGCAGTGATTACATGATAGCGAGCATATATCCCAGCAGTGACAATTGCCAAGAACGGAAATTCGGCATTCGTTGCGCAAAGTGGCGTGGGGCTGATGGTTCGTGGCTTGGCAATAAGGCGTAGAGTGGCAGTTTGTCTCTTCGAGGTTGATGACGTTTTTTGCTTCTTATCTCATTGGGCTGAGTGCGGATGATGAGTTCTTGATGAAGGGGCTGGAAGCGAGGTGGTAGATGCTCAGCATTGCTAGAGAGTGCCTTCTTGTTATTGTTCGGAGAAATCAATAGATCAATATGCTAACTTCCTCGTAAGATTTGTAAAGATTTAAAAATAGAAATGAATGCGAATGCGAATAGCCTAATAAAAAGTCATACGCAATTAAAATTGTAATCGATTCATGATTGTGAATGTCTTGGAACAAATAGGCGATTGGATCATTTTCAACACGCTCAGCATCCTATCATCCACTTTACACAAGACCAGATGAACCATCGAACGCTTCGAAGATACAGCAAGCCAGCATTACCAGGAACCATGACGGTTCTCCAAAGAAGGGTTCTTCAAGCTTCTAGAAGGAATCTATTGCATGATTTGCATGACTAACCGGGATAATTTTGTACTCGTGGCGCAGTAGACTGCCCCTCACCACGGAACTCGGAACATGACTTGCTAATAAACCAACCAGAGTTCTTGTATAAGCCCTATGATCTCCACTCATTGCAATCAGCTATGCCAATTGGGCTTTTAGGCCCTGGATGGCTCGATTCGCTCGCGAGGCTGCAGGTGATTGATTCACTGCCCTAACTGTGCCTCAATCACTACCATAATCCGTGCTGTGCTCTGCCCTATATAAGATCCTCTTACGCACCTTCATCTCAGCCGTTTACTCCTCTGCTTACATCGTTCAGAGATCACATCTCATCTAATTCAACCATCAAACTTGTCTTGAACATCATCAAACAAGATCAAATCTGACGATAAATTTTGAGTAAGAGAACAACTCAAGTAAATCAATTCAACCACA |
| Pfes1 | TTATGAAGGGATTGCTGCAGGCAACGCGGTTTTCATGGGCGACTCTCGAGACGATGAATTACCGGAAGAGGTCTAGATCTAGAAGATGGGAACTACGGCTTGGGCTCGGAAATTCATGACGTGTTGAAAGGCATCGATGAGATGGTGTATTCATCATGATAGAATGGGTGAAAATATTTACTAAAATTAAGCTCGTCGATGAAAGCCATGTTCTAAACCAGGAAATGATATCTAGGGTGATGTTTTTCTGGAACTCTCGTTTTTGAGCTGTGGTATCAGTATGTCTATGTGATTCAATATCATAGCCTCGAGCAGCTTTAATCTGTAAAATGCCCATGTATAATGACTTTGTAGTTGTGAGGAGCGATGACATTGGTACTCGGGAAGATTGACATCTTATACCAACAAGAAAATGAGAACGCATCACGAATCCGGGTGTAGATTGAAATAGACTAGTCTCACATAAGAAAGCATATGATTCCTCTATGAAATGTAGGGAAATGACACTGTAGAACTTATCGCTCGTCATATTTGTAGAAAAAGTAATGGAGTTGTAGATTTGAGTGATCCTTAACGAAGAACAGTAGCCACCTAAGGAAAGGAAGCAAGTTCAAGTTTTGCTCGCCTCGTTCCATGAGTGAATGGCAGTCCGAAGCTGCGGCAATGCCAAACCATCAGGAATAATGTGTCTATGAATAACCAATTGGTCTCATGCAGTGACTTGTGAGATTTGCATGACTATTGGCGTACATGTCGTGAGCGGATTGTTCAGAGGACTCTCTTTAGATTCGTCAACTTCACAAGCTTGAGTTGAGGTCGTATATGCATGACCAATGAGTTCTGAGTACTTGTTTAATGGGTATCAAATTGTAACTTTACATAAGGATACCCAAGATAATCACAGTACGAGGAAGGATGTTTATATTCAAAGTATTCTCCCGTATCAGACTCGAGCAGTTCTTCAAGAGTGTCTCGAGGTAATATGTTGAGATCTCATACATATGTATGTGTTCATGAAGCAACTTCTCAGTCGAATAAGAAATCGAAATGAATATGTAGTAACAGAAGACTGATCTACAATCAAACAAGAATCTTTCATCTATATTCGAGATCGCTAAGAAGGCTGTCTACCAACCAATCGTCTTGACTATCCTCAAGGAAGTGTGTGACTAATCATGTCTTCACCGCCAAAATTCTGCCTTATGCCATGCCCAAAAAAAAGTCCAGATTGGATTGGAAGCATCTCGAAGTTAGTAGACGTAAATAGACAAATTTAATAGTCATCTCATACAAAAGAGAAGAAATCTTTTTAGACACATTTCTATACAACTTACAACTTCTCTTGATCTCATCTACAATTTTCTGAGAATTCAATACTTTCTTGTTAAATATACTATAATCCTTTTCGGCGCAAAGCATTTTCACCTACCGGTTCTCGATTTTCGATCAGACAAATTTCCACAACTTTCAGTTCTAAAGAGAACATAGAAATCTGCCATC |
| Pyqae | CCACTTTTAATATTTTTTTTTAAAATCGATGGTCAATACTTTTTGATTTTGAATTAGCAATGAAATATTACAAAAGCTTATCATTTATAATCTATTTTAATTTGATTTGATTTTTAACTTTTTTTCGAAAAAAAAGTTCAATTTTAAAGCAATAGTGTTTTAAAAAAAGATTTGATTTTGGAGGGTTTGGTATGTCGTTTTTTTTTACATCAACCGCGGACCCCAGGTATTGTACCATGACACCGTTTGTTCTGATGCTCAGCCACATGGCCGACCAAATAGACCAAGCTTTCTTGAATCTTAAATACAGTCTAGACATTGCTTTGACTCGTCTCGAGTAGCCTTATAAGCACCAATACGCACCAATACGCACCAATACAAGTCGACCCGAGAAATGCTAGGATATTTTCTTTGCAGCACACACAACCCAACCTCGTCATTTCACTGTGGCTTTGAATTACAGTTGCCGGGATTCGGAATATCGAGATGATTAATTGGAAGAAATGAATAATCACAAGATGAAGTTGACAGCCAAGATTTCCGAATGTTCCATGTACATATCTCTTGCCGTGTATGCGGCTTAGCAAAGTGTCGATTACAGAGAGAGACGAATCAGATGCCGGGTTTGCAATCACATGAAGATTTTCCGCGAGCAGCCACAGCTCAGCCACAGTGCCACATCTGAAGGTCCCCGAAACCGAAAAAGAAACCAATCGGAATGCATGCGGCACTGCGAAAAGCCAATGTCCTTCCCTCCCGGCAGTTACCCTGATGCTCCTTCCTGCTTTGTGCCCATCCGTTGTAATTGTTGAGTCGTCGCAACATCCACGAGAACCATACAAATGGGGTGACTCGATTGTGGCACACAGGAGTTTTAATATCATGCAGGTTCCTCGAGGGACACTCACCATGCGTCGGATTTAATTGTGTCCATAGGCAGGATTGGCTGGTTGGCGCTTTAGTTTCTCTTGGAAGATAAAAACACGACGACTGAAGCAAGTGAAGGGAGTGAAGGAAGAGATGGAAGAGATATTATGACGTAATGTGGTTTAAGAATCTGCTAGTCACGGACGATGGTTGGCTGGCCTTGTGAATCGAATTTCTTGGTTGTGCCACACCTGTGAATTGGGCTAAGCTGCTTTCGGCCGTGATAATGCCAACTCTTTTCTTGCTGGTTGATAAGAATAGGTAAAATAAGGTAAATTAGTGACGTTGTAATTTCGACTCAAGCAAAATCGGGAAATCGTACCATTCAAGTTCCACACGCCCAAGGCCCAAAGCTCTCCTCAGTGCTCATACATAAAGGTCCACTTTCCCTGTGGTGAGGTACTTCCTTTTGCAAACATACATTCCAATCTTCTCTCCATCAAACATCGATCGTCGATCAGCAACCATCAAGACAAAGAATAAGAACTCTTCGTTTTCCAAGACACCCATCAATCAGTCAGTCGACAACACACTTCGACCACACACACCCTTTCACCAATTAAATCCAACATC |
| Pgdh | ATTAACCACGCATCTCCTCATCTCCCCTCAAAATTACCCCTCATCCGATACCGGAAAAATTCCGATTAACGAAAGTATTCATCACAACATTGACAAATCTTTGTCCCTATCTTCACCAAGAATACTCGAAACATTGGCATCCACCTTGAGCCACCTTCGTCGGATGTATTGTAATGCTTTACGAAGTGTTGAGTCGAGGTAAGCGATTGCCGCAAGAAAATCCATTTGAGGGTGAGGTGGCACGTTGAAGTTGTTACGCTTCTGCAGGAACAAAATGAAGTATTACCAAATATTTTCATGGGTTCAGTTCCAAGACTTCGGGACATGTAGGAATACTGGGAAATCTCGGGGAAATGTACTGGAACGTTTTTATCCTGGCTGCCTGATTTTGAGACGCGGGGCTTTCATTTTTCCATCTCACATCTTCATGAAAGAGTTGATATCCTTGATCATTACCCCAGCGTGGCTAGGGATCTTTGCATGCGCCATACATGCCTCAACATTGCATTTAGAAAAAGTTTGCCGAGCATGCTAATGCAAGATTCCGGGTACGTGAAAGCTTGGGCTGTTAATCTATATACGTACTGACCAATAGGAGATGTACGATAAGAATTTCTATTTGATTCATCGGTGACACCGTACATAGCGTGCCATTGGTCAATTTCAACCTCTCTCCTTCTCTGCTTTCAGTTGGAGTTGAATAATCTTCAAAACGGGACGATTTTCCGTTGTCGGGATTTTTCAGGTATCCAGTAAAAGCAAAGATCCAGAGAAGAGGAGAGAAGGGAAGGGTAAACTTCCTGGAACTAATCTCATCTTGGTTTAGATCAATGGGGGTGGATAAAGCAGAGGATCAATTAGATTTGATATTTCGACATGTCTCCATTGTCCCACGCAGATACACCAAATATCAAGTGGGATGGATGGAAGCTTCAGCCATACAACATGCAATGTAACGATACGACCGATCACGGCCTCGAGGGGGTCACGTCATGTATCAGATTTTCTTCAATTTTCTCGTGGAAGTTGCAATTCCGCTGAGATTGAAGACTCTTGCGGTACAGGCGATCATTTTCATAGTAAAAACAATTATGCAAGAGGTTGAGCGAGACACATTGATTTGACAGGAAGAATGCAGTTGGAAAATCTTTATCAAGTCATCCATCTTCATTACTTTTTTCACCCCTCCTCCCCTCTATCCGATCCTCCAATATCGGAGCCACAAAAAAAATACTGGAGGCATGGATGCACATACAAGCCTTCACCTAGTTAGACAATTAATTAGTACATTTCATCGAATATTCAATGGGTCCTGAACCGGCACCAAAGTGTTACTAGTTCGTAGACTCTAATCCACCCCACCTCGATACAAAAAGGTCTTTCTTCCCTTCAGTCTTGCCGGTTGCTTTACTCAATCTACCAACTTCTATTCTTCTTTTTCACTCGACGTTCATATTGCAAATTTCTTCACTTGATACCCAAACACCCACAATAATAATC |

Table S4 Characteristics of the selected promoters of *B. cinerea*. The putative TSSs of promoters were predicted by Berkeley Drosophila Genome Project.

| Promoter name | Predicted regulatory elements* | |
| --- | --- | --- |
|  | TSS | TATA box |
| Pthi4 | -859 to -908, -196 to -245 | -897 |
| Pef1a | -845 to -894, -304 to -353 | ND |
| Phs70 | -748 to -797 | -1049 |
| Phs90 | -1058 to -1107, -339 to -388 | ND |
| Paba1 | -909 to -958 | -1348 |
| Phy1 | -960 to -1009, -468 to -517, -272 to -321, -196 to -245 | -509 |
| Phy2 | -138 to -187 | -178 |
| Pfes1 | -299 to -250 | ND |
| Pyqae | -624 to -673, -485 to -534, -163 to -212 | -1159 |
| Pgdh | -887 to -936 | ND |

*-1 bp counted from TLS (translation start site, ATG); TSS: transcription start site; TATA box (TATAWAW).

ND is not detectable.

Table S5. FPKM values of key enzymes in the ABA biosynthetic pathway and secondary metabolites in *B. cinerea* at different fermentation time points.

| Gene | Accession  No. | Gene expression level  (FPKM value) | |
| --- | --- | --- | --- |
|  |  | 24h | 96h |
| *Bcerg10* | BCIN_05g07430 | 562.15 | 197.96 |
| *Bcerg13* | BCIN_11g00330 | 165.11 | 56.12 |
| *Bchmgr* | BCIN_05g03150 | 443.83 | 236.79 |
| *Bcerg12* | BCIN_05g03770 | 31.53 | 21.63 |
| *Bcerg8* | BCIN_12g04570 | 249.35 | 254.59 |
| *Bcmvd1* | BCIN_04g01890 | 224.29 | 59.87 |
| *Bcidi1* | BCIN_12g01780 | 263.85 | 132.66 |
| *Bcerg20* | BCIN_15g03120 | 136.53 | 73.47 |
| *Bcaacs* | BCIN_07g02750 | 31.73 | 23.29 |
| *Bcaba1* | BCIN_08g03850 | 7362.01 | 5160.53 |
| *Bcaba2* | BCIN_08g03840 | 1718.58 | 1308.30 |
| *Bcaba3* | BCIN_08g03880 | 8270.41 | 3065.03 |
| *Bcstc1/Bcbot2* | BCIN_12g06390 | 0.36 | 0.22 |
| *Bcstc2* | BCIN_08g02350 | 5.77 | 3.17 |
| *Bcstc3* | BCIN_13g05830 | 0.08 | 0.10 |
| *Bcstc4* | BCIN_04g03550 | 112.57 | 12.53 |
| *Bcphs1* | BCIN_01g04560 | 15.48 | 40.98 |
| *Bcdct1* | BCIN_01g04920 | 0.09 | 0.15 |
| *Bcdct3* | BCIN_08g03560 | 0.24 | 0.20 |
| *Bcpks1* | BCIN_14g00600 | 0.02 | 0.09 |
| *Bcpks2* | BCIN_02g01680 | 0.06 | 0.17 |
| *Bcpks4* | BCIN_11g02700 | 23.33 | 39.07 |
| *Bcpks8* | BCIN_07g02920 | 4.63 | 5.85 |
| *Bcpks11* | BCIN_14g01290 | 0.68 | 1.78 |
| *Bcpks17* | BCIN_03g02010 | 0.10 | 0.28 |
| *Bcpks19* | BCIN_08g00290 | 0.31 | 0.72 |
| *Bcpks20* | BCIN_04g00640 | 1.29 | 1.97 |
| *Bcpks21* | BCIN_05g08400 | 0.68 | 0.52 |
